# Monoclonal antibodies for the S2 subunit of spike of SARS-CoV-1 cross-react with the newly-emerged SARS-CoV-2

**DOI:** 10.2807/1560-7917.ES.2020.25.28.2000291

**Published:** 2020-07-16

**Authors:** Zhiqiang Zheng, Vanessa Marthe Monteil, Sebastian Maurer-Stroh, Chow Wenn Yew, Carol Leong, Nur Khairiah Mohd-Ismail, Suganya Cheyyatraivendran Arularasu, Vincent Tak Kwong Chow, Raymond Tzer Pin Lin, Ali Mirazimi, Wanjin Hong, Yee-Joo Tan

**Affiliations:** 1 Infectious Diseases programme, Department of Microbiology and Immunology, Yong Loo Lin School of Medicine, National University Health System (NUHS), National University of Singapore, Singapore; 2 Immunology programme, Department of Microbiology and Immunology, Yong Loo Lin School of Medicine, National University Health System (NUHS), National University of Singapore, Singapore; 3 Department of Laboratory Medicine, Karolinska Institute, Huddinge, Sweden; 4 Public Health Agency of Sweden, Stockholm, Sweden; 5 Bioinformatics Institute (BII), A*STAR (Agency for Science, Technology and Research), Singapore; 6 Department of Biological Sciences (DBS), National University of Singapore, Singapore; 7 National Public Health Laboratory (NPHL), National Centre for Infectious Diseases (NCID), Singapore; 8 Institute of Molecular and Cell Biology (IMCB), A*STAR (Agency for Science, Technology and Research), Singapore; 9 Department of Microbiology and Immunology, Yong Loo Lin School of Medicine, National University Health System (NUHS), National University of Singapore, Singapore; 10 National Veterinary Institute, Uppsala, Sweden

**Keywords:** coronavirus disease 2019, COVID-19, SARS-CoV-2, spike protein, cross-reactive antibodies

## Abstract

**Background:**

A novel coronavirus, SARS-CoV-2, which emerged at the end of 2019 and causes COVID-19, has resulted in worldwide human infections. While genetically distinct, SARS-CoV-1, the aetiological agent responsible for an outbreak of severe acute respiratory syndrome (SARS) in 2002–2003, utilises the same host cell receptor as SARS-CoV-2 for entry: angiotensin-converting enzyme 2 (ACE2). Parts of the SARS-CoV-1 spike glycoprotein (S protein), which interacts with ACE2, appear conserved in SARS-CoV-2.

**Aim:**

The cross-reactivity with SARS-CoV-2 of monoclonal antibodies (mAbs) previously generated against the S protein of SARS-CoV-1 was assessed.

**Methods:**

The SARS-CoV-2 S protein sequence was aligned to those of SARS-CoV-1, Middle East respiratory syndrome (MERS) and common-cold coronaviruses. Abilities of mAbs generated against SARS-CoV-1 S protein to bind SARS-CoV-2 or its S protein were tested with SARS-CoV-2 infected cells as well as cells expressing either the full length protein or a fragment of its S2 subunit. Quantitative ELISA was also performed to compare binding of mAbs to recombinant S protein.

**Results:**

An immunogenic domain in the S2 subunit of SARS-CoV-1 S protein is highly conserved in SARS-CoV-2 but not in MERS and human common-cold coronaviruses. Four murine mAbs raised against this immunogenic fragment could recognise SARS-CoV-2 S protein expressed in mammalian cell lines. In particular, mAb 1A9 was demonstrated to detect S protein in SARS-CoV-2-infected cells and is suitable for use in a sandwich ELISA format.

**Conclusion:**

The cross-reactive mAbs may serve as useful tools for SARS-CoV-2 research and for the development of diagnostic assays for COVID-19.

## Introduction

The severe acute respiratory syndrome coronavirus (SARS-CoV-1), a virus considered to have a zoonotic origin, is the aetiological agent for the infectious disease, SARS, which first emerged in 2002–2003 [1,2]. In December of 2019, another novel coronavirus (SARS-CoV-2), which causes coronavirus disease (COVID-19), appeared to have crossed species barriers to infect humans and was effectively transmitted from person to person, leading to an outbreak in Wuhan, China [3-5]. This virus subsequently spread worldwide, leading the World Health Organization (WHO) to declare a pandemic on 11 March 2020 [6]. To date, SARS-CoV-2 continues to pose a high global health and economy burden, and as at 3 May 2020, COVID-19 had affected 215 countries with over 3.35 million confirmed cases.

To tackle the problems caused by SARS-CoV-2, improving its detection and knowledge of its infection mechanism is important. In this respect, the viral surface spike glycoprotein (S protein) has been demonstrated to play key role in host cell selectivity and binding. The S protein is functionally divided into two subunits, with the S1 subunit containing the receptor binding domain (RBD), which allows attachment to host cells, and the S2 subunit mediating fusion between viral and host membranes (reviewed by Li, F.) [7].

Phylogenetic analysis revealed that like SARS-CoV-1 and bat-derived SARS-like coronaviruses (SL-CoVs), SARS-CoV-2 belongs to lineage B of the betacoronavirus genus [8,9]. A study of 56 complete and partial SARS-CoV-2 genomes isolated from COVID-19 patients showed very high sequence conservation of more than 99%, indicating a recent introduction of the virus into the human population [10]. Although the animal source of SARS-CoV-2 is not clear, SARS-CoV-1 is believed to have originated from SL-CoVs residing in bats [11-14]. For the majority of SL-CoVs, the S1 subunit has low sequence identity to that of SARS-CoV-1, which suggests species-dependent receptor binding [14,15]. On the other hand, the high amino acid sequence identity of more than 90% in the S2 subunit suggests that the fusion mechanism during virus infection is well-conserved [14,15].

While SARS-CoV-2 shares higher whole-genome sequence identity with bat-SL-CoVZC45 and bat-SL-CoVZXC21 (88–89%) than with SARS-CoV-1 (79–82%), the RBD of SARS-CoV-2 is more similar to SARS-CoV-1 RBD [8,9]. In line with this, several research groups have demonstrated that SARS-CoV-2 utilises the same host receptor, angiotensin-converting enzyme 2 (ACE2), as SARS-CoV-1 for viral entry [3,16-18]. Due to its role in virus entry, the S protein has been the target for the generation of monoclonal antibodies (mAb). 

In our previous work, we used five different fragments of SARS-CoV-1 S protein to immunise rabbits. A fragment corresponding to residues 1029 to 1192 in the S2 subunit of SARS-CoV-1 was found to stimulate neutralising antibodies against SARS-CoV-1 [19]. This fragment was subsequently used to generate a panel of murine mAbs with their respective binding domains characterised and described in Lip et al. [20]. One of them, mAb 1A9, which binds to the S protein through a recently identified epitope within the S2 subunit at amino acids 1111–1130, has the ability to bind and cross-neutralise pseudotyped viruses expressing the S protein of human SARS-CoV-1, civet SARS-CoV and bat SL-CoV strains [21]. In this study, we aim to verify if the sequence of the immunogen used to generate mAb 1A9, as well as three other mAbs, is conserved in different coronaviruses and if these mAbs bind to the S protein of SARS-CoV-2 expressed in mammalian cell lines. Importantly, mAb 1A9 is investigated for its ability to detect the S protein in SARS-CoV-2 infected cells and purified S protein in a sandwich ELISA format when paired with another mAb binding to the S1 subunit of SARS-CoV-2.

## Methods

### Cells

Vero E6 and COS-7 cells were purchased from the American Type Culture Collection (Manassas, VA, United States) and cultured in Dulbecco’s Modified Eagle’s Medium (DMEM; Thermo Fisher Scientific, Waltham MA, United States) supplemented with 10% fetal bovine serum (FBS; HyClone, Logan, UT, United States), 100 units/mL penicillin and 100 µg/mL streptomycin (Thermo Fisher Scientific). 293FT cells were purchased from Invitrogen (Carlsbad, CA, United States) and grown in DMEM supplemented with 10% FBS, 100 units/mL penicillin, 100 µg/mL streptomycin and 500 µg/mL geneticin (Thermo Fisher Scientific). Cells were maintained at 37 °C with 5% CO_2_.

### Purification of monoclonal antibody 1A9

The hybridoma for mAb 1A9 was previously generated [20]. All mAbs were purified from cell culture supernatants using HiTrap protein G HP affinity columns (GE Healthcare, Chicago, IL, United States) and stored at −80 °C. The purity of the mAb was confirmed by sodium dodecyl sulphate-polyacrylamide gel electrophoretic (SDS-PAGE) analysis. The concentration of the purified mAb was determined using the Coomassie Plus protein assay reagent (Thermo Fisher Scientific).

### Plasmids for expression of full spike protein and fragments

SARS-CoV-2 S-protein-expressing plasmids were codon-optimised and generated by gene synthesis (Bio Basic Asia Pacific, Singapore) according to GenBank accession number: QHD43416.1. One plasmid is for expressing untagged full-length S protein while the other is for expressing a Myc-tagged S-protein fragment consisting of residues 1048–1206 (SARS-CoV-2 numbering). The pXJ40-Myc expression vector was used as an empty vector control and pXJ40-Myc-HBcAg plasmid expressing Myc-tagged hepatitis B virus core antigen (HBcAg) was used as a negative control.

### Transient transfection and western blot analysis

293FT cells were seeded onto 6-cm dishes 24 hours before transient transfection using X-tremeGENE HP DNA transfection reagent (Roche, Basel, Switzerland) according to the manufacturer’s protocol. At 24 hours post-transfection, cells were harvested, spun down by centrifugation and washed with cold phosphate buffered saline (PBS) twice. Cells were then resuspended in 2× Laemmli sample buffer, boiled and sonicated. Clarified supernatant containing the protein of interest was obtained by spinning down the cell lysate at 13,000 rpm at 4 °C to remove the cell debris and further analysed by western blot (WB) analysis. Equal amounts of total cell lysates were loaded per lane and resolved using electrophoresis on SDS-PAGE gels and transferred onto nitrocellulose membrane (Bio-Rad, Hercules, CA, United States). The membrane was blocked in 5% skimmed milk in Tris-buffered saline with 0.05% Tween 20 (TBST) for 1 hour at room temperature (RT) and incubated with primary antibodies at 4 °C overnight. After the membrane was washed with TBST, it was incubated with a horseradish peroxidase (HRP)-conjugated secondary antibody (Thermo Fisher Scientific) at RT for 1 hour. The membrane was then washed with TBST again and bound antibodies visualised with enhanced chemiluminescence substrate (Thermo Fisher Scientific) using ChemiDoc MP Imaging System (Bio-Rad).

### Transient transfection and immunofluorescence analysis

For immunofluorescence (IF) analysis, COS-7 cells on glass coverslips were transfected as above and fixed at 24 hours post-transfection in 4% paraformaldehyde for 10 min at RT followed by permeabilisation with 0.2% Triton X-100 (Sigma-Aldrich, St. Louis, MO, United States) for 5 min. Fixed cells were then blocked with PBS containing 10% FBS for 30 min at RT. Cells were immunolabelled for 1 hour at RT with the indicated murine mAb and 45 min with Alexa Fluor 488-conjugated goat anti-mouse IgG antibody (Life Technologies, Carlsbad, CA, United States). Immunolabelled coverslips were counterstained with 4′,6-diamidino-2-phenylindole (DAPI; Sigma-Aldrich), and mounted using ProLong Gold Antifade Mountant (Molecular Probes, Eugene, OR, United States). Images were acquired with Olympus CKX53 microscope using Olympus (Tokyo, Japan) LCAch N 20×/0.40 iPC objective lens and Olympus DP27 colour camera with Olympus cellSens software. Each channel was collected separately, with images at 1024 × 1024 pixels.

### ELISA

Whole ectodomain of SARS-CoV-2 S protein with His Tag (Sino Biological Inc., Beijing, China; catalogue number: 40589-B08V1) was diluted with coating buffer (0.1 M NaHCO_3_, 34 mM Na_2_CO_3_) and a total of 20 ng of protein was loaded into individual wells of a 96 well plate (Nunc, Roskilde, Denmark) and allowed to coat overnight at 4 °C. Plates were then washed four times with 0.05% Tween 20 in PBS (PBST) and blocked with 5% bovine serum albumin (BSA)/PBST for 30 min before murine antibodies serially diluted with blocking buffer were added to desired wells for 1 hour. Plate were washed four times with PBST before incubation for 1 hour with HRP-conjugated goat anti-mouse IgG (Thermo Fisher Scientific) secondary antibodies diluted in blocking buffer, and washed four times with PBST. Visualisation of bound secondary antibodies was done by the addition of 3,3',5,5'-tetramethylbenzidine (TMB) substrate (Thermo Fisher Scientific) for 5 min in the absence of light and the reaction was stopped with 2 M sulphuric acid. Optical density at 450 nm (OD450nm) was determined by a Tecan (Männedorf, Switzerland) Infinite M1000 reader and normalised OD450nm was obtained by subtracting background absorbances determined in BSA coated wells.

### Production of monoclonal antibody CR3022

The human mAb CR3022 was expressed in a similar manner as previously described [22]. The variable heavy (VH; GenBank accession number: DQ168569) and variable light (VL; GenBank accession number: DQ168570) genes of CR3022 were generated by gene synthesis (Bio Basic Asia Pacific) and cloned into pFUSEss-CHIg-hIgG1 and pFUSE2ss-CLIg-hK cloning vectors (InvivoGen, San Diego, CA, United States) respectively. Transfection of suspension FreeStyle 293 cells (Thermo Fisher Scientific) and purification of antibodies by fast protein liquid chromatography is as described in our previous study [23].

### Sandwich ELISA

Mab 1A9 was diluted with coating buffer (0.1 M NaHCO_3_, 34 mM Na_2_CO_3_) and 0.1 µg of antibody was coated onto individual wells of a Maxisorp flat-bottom plate (Nunc) overnight at 4 °C. The plate was washed three times with PBST before blocking was done using 5% BSA/PBST at 37 °C for 60 min. Dilutions of His-tagged full length SARS-CoV-2 S protein (Sino Biological Inc., catalogue number: 40589-B08V1) and His-tagged H7N7-HA (Sino Biological Inc., catalogue number: 11082-V08B) were added to desired wells and incubated at 37 °C for 90 min followed by three washes with PBST. 100 µL of CR3022 antibody was added at a concentration of 1 µg/mL and incubated at 37 °C for 60 min followed by three PBST washes before HRP-conjugated goat anti-human IgG (Thermo Fisher Scientific) was added for 60 min at 37 °C. Finally, after three PBST washes, TMB (Sigma-Aldrich) was added for 5 min and the reaction was stopped by 2 M sulphuric acid. The OD450nm was determined by a Tecan Infinite M1000 reader. Statistical analyses were performed using an unpaired, one-tailed Student’s t-test with Welch’s correction for unequal variances. p values < 0.05 were considered statistically significant.

### Virus infection and immunofluorescence

All works with live virus were performed in the biosafety level (BSL)3 facility at the Public Health Agency of Sweden. Vero-E6 cells were infected with SARS-CoV-2 (SARS-CoV-2-Iso/01/human/2020/SWE; GenBank accession number: MT093571) at a multiplicity of infection (MOI) of one in DMEM 2% FBS (Thermo Fisher Scientific). At 24 hour post-infection, cells were fixed with chilled methanol/acetone and the cells were kept at −20 °C overnight. Cells were then stained using mAb 1A9 at 5 µg/mL at 37 °C for 30 min in IF buffer (BSA 0.2%, Triton ×100 0.1% in PBS, pH 7.4). The cells were washed three times with PBS and incubated, subsequently with Alexa Fluor 488-conjugated goat anti-mouse IgG antibody (Thermo Fisher Scientific) in IF buffer containing DAPI for an additional 30 min. Cells were washed three times with PBS before visualisation and image acquisition with fluorescent microscopy. 

### Bioinformatics analysis

S protein reference sequences for SARS-CoV-1, SARS-CoV-2, batRaTG13, Middle East respiratory syndrome (MERS) and human common-cold coronaviruses 229E, NL63, OC43 and HKU1 were downloaded from the National Center for Biotechnology Information (NCBI). A multiple sequence alignment was created with multiple alignment using fast Fourier transform (MAFFT) using the slow but accurate L-INS-I parameter settings [24] and the alignment curated, cut to the target region 1029–1192 (SARS-CoV-1 numbering) and visualised with Jalview [25]. We used Molecular Evolutionary Genetics Analysis (MEGA) X [26] to calculate the number of amino-acid differences for all sequence pairs in the alignment of the mAb target region and the full S protein normalised by the length of the aligned sequence of the respective reference protein to obtain per cent amino acid identities.

To determine SARS-CoV-2 sequence diversity in the S protein within the current pandemic, 230 human and environmental viral sequences were downloaded from GISAID’s EpiCoV database on 1 March 2020. We gratefully acknowledge the authors, originating and submitting laboratories of the sequences on which this part of the research is based. The list is detailed in Supplementary Table 1. The nt sequences were searched with basic local alignment search tool (BLAST)X against the reference S protein. 174 hits covered the full length of the S protein and amino-acid mutations were counted and tabulated using a custom Perl script (Supplementary Table 2).

### Ethical statement

Ethical approval was not required for this study.

## Results

### An immunogenic domain in the S2 subunit of SARS-CoV-1 is highly conserved in SARS-CoV-2 but not in MERS and common cold HCoV

**Figure 1:**
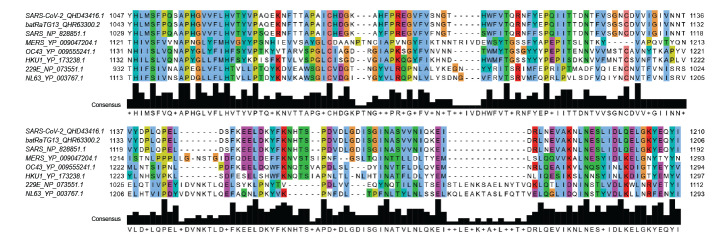
Multiple sequence alignment for the S2 subunit fragment of SARS-CoV-1 spike glycoprotein with other relevant coronaviruses

MERS: Middle East respiratory syndrome; SARS: severe acute respiratory coronavirus 1; SARS-CoV-2: severe acute respiratory coronavirus 2.

Sequence alignment of the S2 fragment corresponding to residues 1029 to 1192 shows that this fragment, which encompasses the heptad repeat (HR)2 but not HR1, is highly conserved in SARS-CoV-1 and SARS-CoV-2 (Figure 1). When compared with additional reference sequences from bat RaTG13 (closest bat precursor), MERS and human common cold coronaviruses 229E, NL63, OC43 and HKU1 (Figure 1), it becomes apparent that the amino-acid identity between SARS-CoV-2 and SARS-CoV-1 is much higher in this region (93%, Table) than over the full protein length (78%, Table) and the similarity drops sharply (< 40% in this region) when considering MERS and the other coronaviruses infecting humans regularly.

The name of the viruses, for which sequences are being compared figure on the left side of the alignment, together with the respective sequences’ GenBank accession numbers.

Colour schemes represent the following categories of amino acids: blue – hydrophobic, cyan – aromatic, green – polar, magenta – negative charge, orange – glycines, pink – cysteines, red – positive charge, yellow – prolines, white – unconserved.

**Table:** Pairwise amino-acid identity across relevant coronaviruses in the sequence fragment of the spike glycoprotein S2 subunit recognised by monoclonal antibody 1A9 or the sequence of the full spike glycoprotein

Query/reference	Pairwise amino-acid identity (%)							
	SARS-CoV-2	BatRaTG13	SARS-CoV-1	MERS	OC43	HKU1	229E	NL63
Fragment region of spike S2								
SARS-Co-V2	100.00	SB	SB	SB	SB	SB	SB	SB
BatRaTG13	99.40	100.00	SB	SB	SB	SB	SB	SB
SARS	93.10	92.50	100.00	SB	SB	SB	SB	SB
MERS	39.00	39.00	39.00	100.00	SB	SB	SB	SB
OC43	39.00	39.00	38.40	51.20	100.00	SB	SB	SB
HKU1	32.70	32.70	30.80	50.60	68.40	100.00	SB	SB
229E	30.80	30.20	32.10	31.50	29.70	30.40	100.00	SB
NL63	30.80	30.20	30.20	32.10	31.60	33.50	64.20	100.00
Full spike protein								
SARS-CoV-2	100.00	SB	SB	SB	SB	SB	SB	SB
BatRaTG13	97.70	100.00	SB	SB	SB	SB	SB	SB
SARS-CoV-1	77.80	78.20	100.00	SB	SB	SB	SB	SB
MERS	35.40	35.40	35.20	100.00	SB	SB	SB	SB
OC43	37.30	37.10	36.90	39.50	100.00	SB	SB	SB
HKU1	35.20	35.30	35.00	39.00	67.00	100.00	SB	SB
229E	41.70	41.50	41.80	41.80	43.50	43.50	100.00	SB
NL63	36.30	36.20	36.20	35.40	39.70	37.80	64.70	100.00

MERS: Middle East respiratory syndrome; SARS-CoV-1: severe acute respiratory coronavirus; SARS-CoV-2: severe acute respiratory coronavirus; SB: shown below. High to low pairwise amino-acid identity are coloured coded respectively by contrasting green to red backgrounds.

The sequence identity is not affected by the order in which paired sequences are compared so only one-way comparisons are shown to avoid redundancies; the abbreviation ‘SB’ is used when the pairwise amino-acid identity in question is already shown in a further cell of the table.

We also studied the sequence diversity across 174 SARS-CoV-2 S proteins derived from nt sequences shared via the GISAID platform [27]. Only four amino-acid mutations were found within the putative antibody-binding region compared with 30 mutations over the full length protein (Supplementary Table 2). Two of these four amino-acid mutations are from a sequence flagged in GISAID’s EpiCoV database as lower quality due to many undetermined bases.

### Four murine monoclonal antibodies bind to a fragment of the spike protein of SARS-CoV-2

**Figure 2:**
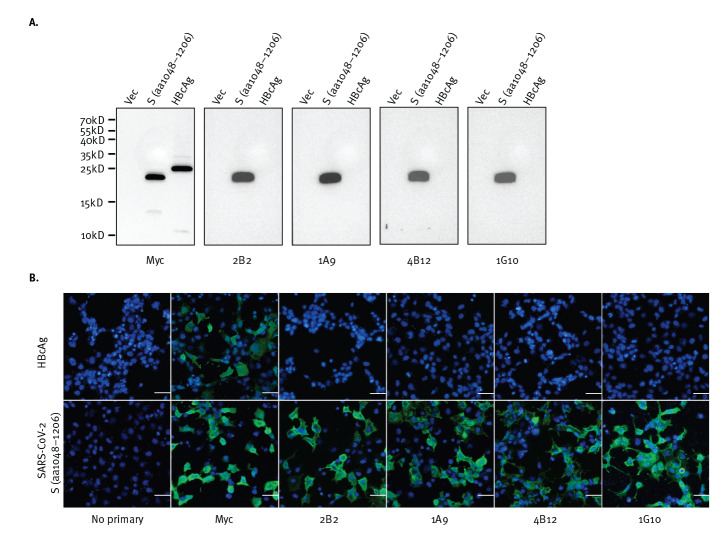
Monoclonal antibodies expected to target a SARS-CoV-2 S protein S2 fragment, (A) hybridise to the peptide fragment in western blot and (B) recognise cells expressing the peptide as shown by immunofluorescence

Four mAbs with distinct binding profiles to SARS-CoV-1, as previously mapped by internal deletion mutagenesis study, were selected for testing to determine if they cross-react with SARS-CoV-2. A fragment containing residues 1048 to 1206 of SARS-CoV-2 S protein was expressed in 293FT cells via transient transfection and WB analysis was performed using the four mAbs, namely 2B2, 1A9, 4B12 and 1G10. As shown in Figure 2A, all four mAbs detected this fragment of SARS-CoV-2, which is consistent with the sequence alignment shown in Figure 1. Due to the easy detachment of 293FT cells, COS-7 cells were used for IF assay instead. IF analysis performed on transiently transfected COS-7 cells showed binding of the four mAbs to this S protein fragment of SARS-CoV-2 (Figure 2B). These interactions are also specific for the SARS-COV-2 S protein (1048–1206) fragment as all four mAbs did not show binding to the negative control HBcAg.

aa: amino acid; HBcAg: hepatitis B virus core antigen; DAPI: 4′,6-diamidino-2-phenylindole; SARS-CoV-2: severe acute respiratory syndrome coronavirus 2; S protein: spike protein; vec: pXJ40-Myc expression vector used as an empty vector control.

A. Each photo depicts a western blot using the primary antibody indicated below it (Myc, 2B2, 1A9, 4B12, or 1G10). Empty vector-transfected 293FT cell products are on the lanes labelled as ‘Vec’, while products of 293FT cells expressing Myc-tagged SARS-CoV-2 S protein fragment (aa 1048–1206; SARS-CoV-2 numbering) or Myc-tagged HBcAg are on the lanes respectively labelled ‘S’ or ‘HBcAg’. Primary antibodies were labelled with horseradish peroxidase-conjugated secondary antibodies. A ladder indicative of the molecular weights in kD of the proteins relative to their vertical position on the blots, is indicated on the left of the panel.

B. Each photo depicts an immunofluorescence assay using either no primary antibody, or the primary antibody indicated below it (Myc, 2B2, 1A9, 4B12, or 1G10). Immunofluorescence assay results of COS7 cells expressing Myc-tagged HBcAg (top photos) or a Myc-tagged fragment (aa1048–1206) of SARS-CoV-2 S protein (bottom photos) using the indicated primary antibodies, followed by Alexa Fluor 488-conjugated secondary antibody (green) are shown. Cell nuclei were counterstained with DAPI (blue). Scale bar = 50 µm.

### Four murine monoclonal antibodies bind to the full-length S protein of SARS-CoV-2

**Figure 3:**
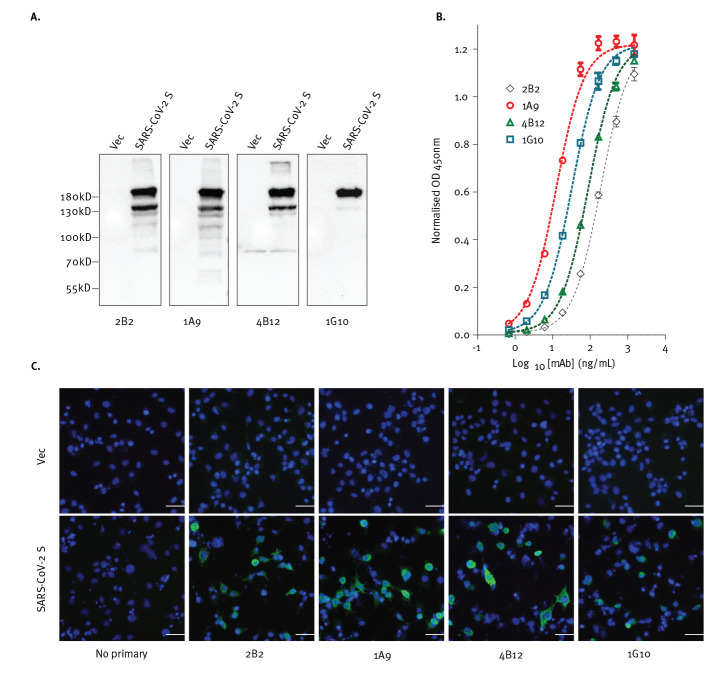
Antibodies expected to target SARS-CoV-2 S protein, (A) hybridise to the denatured protein in western blot, (B) bind to the protein in ELISA and (C) recognise cells expressing the protein as shown by immunofluorescence

DAPI: 4′,6-diamidino-2-phenylindole; mAb: monoclonal antibody: OD: optical density; SARS-CoV-2: severe acute respiratory syndrome coronavirus 2; S protein: spike protein; vec: pXJ40-Myc expression vector used as an empty vector control.

Next, the full-length S protein of SARS-CoV-2 was overexpressed in 293FT and COS-7 cells and detected with each of the mAbs using WB and IF analyses. As shown in Figure 3, all four mAbs bound to the full-length S protein of SARS-CoV-2 (Figure 3A).

A. Each photo depicts a western blot using the primary antibody indicated below it (2B2, 1A9, 4B12, or 1G10). Empty vector-transfected 293FT cell products are on the lanes labelled as ‘Vec’, while products of 293FT cells expressing full-length SARS-CoV-2 S protein are on the lanes labelled ‘SARS-CoV-2 S’. Primary antibodies were labelled with horseradish peroxidase-conjugated secondary antibodies. A ladder indicative of the molecular weights in kD of the proteins relative to their vertical position on the blots, is indicated on the left of the panel.

B. The abilities of 2B2, 1A9, 4B12 and 1G10 monoclonal antibodies to bind to SARS-CoV-2 S protein was determined by ELISA. Individual wells were coated with 20 ng of SARS-CoV-2 S protein and incubated with serially diluted mAbs as indicated. A representative plot from three independent experiment is show for each antibody and error bars correspond to standard deviations of each mAb experiment carried out in triplicates.

C. Each photo depicts an immunofluorescence assay using either no primary antibody, or the primary antibody indicated below it (Myc, 2B2, 1A9, 4B12, or 1G10). Immunofluorescence analysis was performed on empty vector-transfected COS-7 cells (top photos) and cells expressing full-length SARS-CoV-2 S protein (bottom photos). The indicated primary antibodies were used followed by Alexa Fluor 488-conjugated secondary antibody (green). Cell nuclei were counterstained with DAPI (blue). Scale bar = 50 µm.

The binding of these mAbs to recombinant purified S protein was also determined using indirect ELISA where different concentrations of antibodies were used for binding. Binding to S protein was observed for all four mAbs with 1A9 showing the strongest binding (Figure 3B). Similarly, all four mAbs bound to the full-length S protein of SARS-CoV-2 when tested via IF (Figure 3C). Collectively our data demonstrates the ability of all four mAbs to bind full-length S protein in both its native and denatured forms.

### Utility of monoclonal antibody 1A9 for detection of S protein in a sandwich ELISA format and in SARS-CoV-2 infected cells

**Figure 4:**
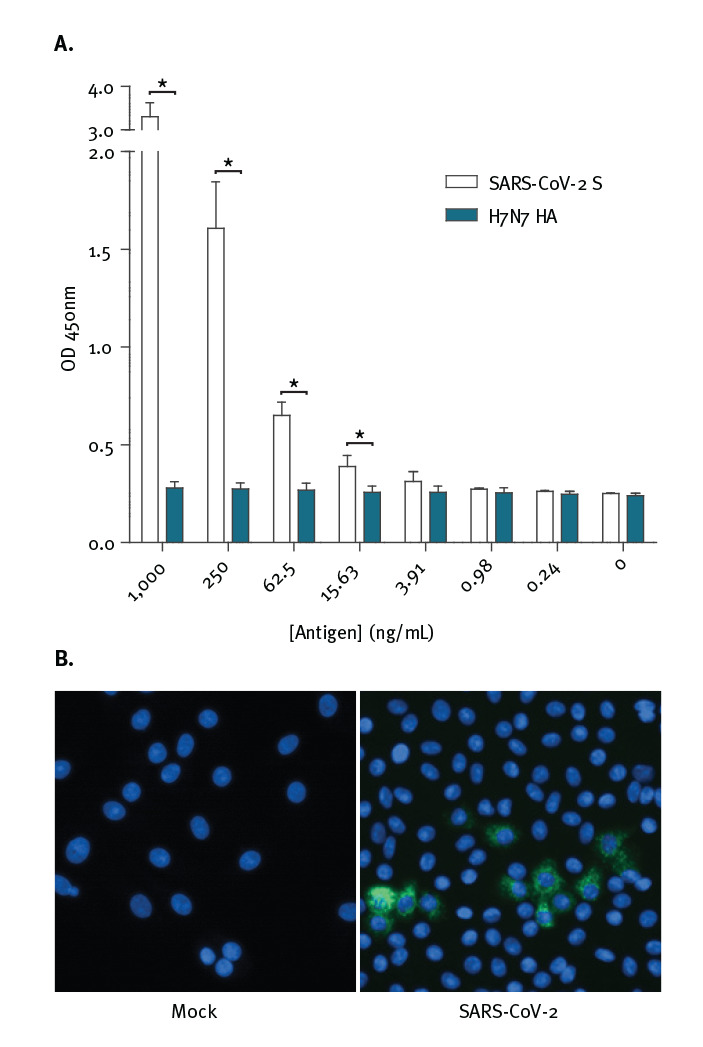
Performance of monoclonal antibody 1A9 for detection of (A) S protein in a sandwich ELISA format and (B) SARS-CoV-2 infected cells

HA: haemagglutinin; H7N7: influenza A (H7N7); mAb: monoclonal antibody; MOI: multiplicity of infection; OD: optical density; SARS-CoV-2: severe acute respiratory syndrome coronavirus 2.

Based on indirect ELISA data, mAb 1A9 has the strongest binding to S protein when compared with the other three mAbs. Hence, a sandwich ELISA was performed to determine if it can be paired with the human mAb CR3022 which is known to bind to the S1 subunit of SARS-CoV-2. As shown in Figure 4A, recombinant S protein was detected at 15.6 ng/mL and above when 1A9 was used as a capture antibody and CR3022 was used as a detector antibody. Since the S protein was His-tagged, a His-tagged haemagglutinin (HA) protein of influenza A virus was used to check for specificity of binding. The absorbance readings in the presence of S protein were significantly higher than that in the presence of HA for protein concentrations of 15.6 ng/mL and above.

A. Sandwich ELISA assay to determine mAb 1A9 ability to pair with the human mAb CR3022 for the detection of a His-tagged SARS-CoV-2 spike protein. 1A9 and CR3022 were used as capture and detector antibodies respectively. His-tagged HA protein of influenza A (H7N7) virus was used as a negative control. Averaged readings across three replicate experiments are presented. Error bars represent standard deviations across the three replicate experiments. Asterisks indicate significantly increased binding of the antibody pairs to SARS-CoV-2 S protein compared to influenza A (H7N7) HA at p < 0.05.

B. Vero E6 cells were mock-infected (left panel) or infected with SARS-CoV-2 (right panel; MOI of 1). At 24 hour post infection, the cells were stained with mAb 1A9 (5 µg/mL) followed by Alexa Fluor 488-conjugated secondary antibody (green). Nuclei were counterstained with 4′,6-diamidino-2-phenylindole (DAPI; blue).

Next, 1A9 was tested on SARS-CoV-2-infected Vero-E6 cells. As shown in Figure 4B, mAb 1A9 stained a considerable number of SARS-CoV-2-infected cells at 24 hours post-infection showing that it is sensitive enough to detect the expression of S protein during infection.

## Discussion

Numerous mAbs against the S protein of SARS-CoV-1 have been generated for research and diagnostic assay development. Some of these may be able to cross-react with the S protein of SARS-CoV-2 and serve as tools to aid research on this newly emerged virus. In this current study, an immunogenic domain in the S2 subunit of SARS-CoV-1 was found to be highly conserved in multiple strains of SARS-CoV-2 (Figure 1 and Table). Consistently, WB and IF analyses showed that four different mAbs generated using this SARS-CoV-1 domain were cross-reactive against the S protein of SARS-CoV-2 (Figures 2 and 3).

Recent cross-reactivity studies have evaluated SARS-CoV-1 neutralising antibodies that bind to the RBD-containing S1 subunit. Although both SARS-CoV-1 and SARS-CoV-2 use ACE2 as a receptor for viral entry [3,16], several SARS-CoV-1 RBD-directed mAbs did not cross-react with SARS-CoV-2 RBD [28,29]. Interestingly, CR3022, which was isolated from a SARS convalescent patient [22], showed cross-reactivity to SARS-CoV-2 RBD and recognises an epitope that does not overlap with the ACE2 binding site [28]. Among the four mAbs tested in this study, indirect ELISA showed that 1A9 binds strongest to the S protein of SARS-CoV-2 (Figure 3B). To determine if 1A9 is useful for detection of S protein in a sandwich ELISA, it was paired with CR3022 since 1A9 binds to S2 subunit while CR3022 binds to S1 subunit. As would be expected, these two antibodies can be paired to detect S protein from 15.6 ng/mL (Figure 4A). In addition, mAb 1A9 stained a considerable number of SARS-CoV-2-infected cells at 24 hours post-infection showing that it is sensitive enough to detect the expression of S protein during infection (Figure 4B). Thus, mAbs 1A9 will be useful for studying the kinetics of SARS-CoV-2 replication in vitro and development of diagnostic assays for COVID-19. It is noteworthy that cytotoxic T-lymphocyte (CTL) epitopes also reside at residues 884–891 and 1116–1123 within the S2 subunit of SARS-CoV-1 [30]. Interestingly, the latter CTL epitope overlaps with the epitope recognised by mAb 1A9 [21]. Hence, the S2 subunit may serve as an important antigen for inducing both humoral as well as cell-mediated immunity against SARS-CoV-1 and SARS-CoV-2.

To our knowledge, this is the first study showing that mAbs targeting the S2 domain of SARS-CoV-1 can cross-react with SARS-CoV-2 and this observation is consistent with the high sequence conservation in the S2 subunit. The ability of these antibodies, particularly 1A9, to detect SARS-CoV-2 S protein in indirect and sandwich ELISAs demonstrate their utility for detection of SARS-CoV-2 infections in a public health setting. Whether or not the current sensitivity of these antibodies are sufficient for robust detection of SARS-CoV-2 infections in a clinical setting and how they compare to existing PCR-based detection remains to be determined. Successful development of these antibodies into a point of care diagnostic kit will provide a complementary approach to existing detection methods. Besides the mAbs characterised here, several other mAbs have been reported to bind to epitopes in the S2 subunit of SARS-CoV-1 [31-33]. Thus, it will be important to determine if these mAbs can also cross-react with SARS-CoV-2.

## Supplementary Data

21000 Supplementary Table 1

## Supplementary Data

79000 Supplementary Table 2
